# Both parents migrating and left-behind children’s cognitive ability in rural China: does it pay?

**DOI:** 10.3389/fpubh.2024.1370436

**Published:** 2024-04-17

**Authors:** Mengzhu Yao

**Affiliations:** China Economics and Management Academy, Central University of Finance and Economics, Beijing, China

**Keywords:** left-behind children, migrating parents, cognitive achievement, direct and indirect impact, China

## Abstract

**Introduction:**

While the well-documented negative correlation between both parents migrating and the academic performance of left-behind children (LBC) in rural China is widely acknowledged, it’s important to recognize that statistical data reveals millions of children experiencing both parents migrating. This discrepancy between the documented negative impact and the prevalence of both parents migrating can be attributed to previous studies primarily focusing on the direct effects

**Methods:**

Employing national representative panel data and FE model, this study estimates the direct impact of both parents migrating and the indirect effects of both parents migrating through private tutoring, family tutoring, family income, and boarding school participation. Finally, we consolidate the direct and indirect impacts to determine whether both parents migrating has a positive or negative net effect on LBC’s cognitive ability.

**Results:**

The direct effect of both parents migrating on LBC’s standardized cognitive ability is -0.140, indicating a negative direct impact of both parents migrating on LBC’s cognitive ability. However, the indirect effects of both parents migrating through private tutoring, family tutoring, family income, and boarding school participation are -0.017, -0.008, 0.306 and 0.119 respectively. The toal effect of both parents migrating on LBC’s standardized cognitive ability is 0.260.

**Conclusion:**

The initially observed negative direct impact of both parents’ migrating can be completely offset by the indirect impact channels, including private tutoring, family tutoring, family income, and boarding school participation. In contrast to prior research, this study unveils a positive overall impact of both parents’ migration on LBC’s school performance.

## 1 Introduction

The negative correlation between both parents migrating and the academic performance of left-behind children (LBC) in rural China has been well-documented. For instance, based on data collected in Hunan province, Zhang et al. (1) demonstrated that being left-behind by both parents has a detrimental impact of over 5% on children’s cognitive development. Similar findings have been reported by Zhou et al. (2) using data from children left behind in Anhui and Jiangxi provinces, as well as Zhao et al. (3) using data collected in Qinghai province and the Ningxia autonomous region in China.

However, statistical data reveals that a substantial number of children in rural China experience both parents migrating. According to the Ministry of Civil Affairs (4), there were more than 9 million left-behind children with both parents migrating in rural China. Data from other sources indicate even larger numbers. For example, Tao and Zhou (5) revealed that there were 30 million LBC with both parents migrated in 2005. Similarly, both Zhang et al. (1) and Zhou et al. (2) found that the percentage of children with both parents migrating was about 40% in rural, surpassing the percentage of children with only one parent absent and children with all parents at home.

The inconsistency between the documented negative impact and the prevalence of both parents migrating may be attributed to previous studies primarily addressing the direct effects, with some exceptions such as Tao and Zhou (5) and Chen et al. (6). Using data collected in Anhui and Jiangxi provinces, Tao and Zhou (5) demonstrated that the additional positive impact of both parents migrating, such as increased family income, is outweighed by the negative impact, such as the reduction in after-school family tutoring, resulting in a net negative impact on LBC’s academic performance. Similarly, Chen et al. (6) also concluded that, when considering the impact of family income and after-school family tutoring, the net effect of both parents migrating on children’s academic performance is negative. This negative net impact contradicts the reality of a large number of migrants leaving their children in their hometown, as previous studies have indicated that children’s education is one of the major factors influencing return migration (7, 8). Moreover, the contradiction becomes even more apparent when non-cognitive factors are considered (9).

The objective of this study is to comprehensively analyze the overall impact of both parents migrating on children’s academic performance. Specifically, this study will initially estimate the direct impact of both parents migrating on LBC’s academic performance. Subsequently, we will assess the indirect effects through which both parents migrating influences children’s academic performance, including factors like family income, boarding school attendance, private tutoring, and family tutoring. Finally, we will consolidate the direct and indirect impacts to determine whether both parents migrating has a positive or negative net effect on LBC’s academic performance. By doing so, we aim to minimize estimation biases and provide a more accurate answer to this important question.

This study focuses on the impact of both parents migrating on the academic performance of LBC for two key reasons. First, the impact of the migration of a single parent may be relatively insignificant. For example, research conducted by Zhang et al. (1) and Zhou et al. (2) indicates that while the migration of both parents negatively affects children’s cognitive development, the impact of being left behind by one parent is less significant. These studies also revealed that only when both parents are absent is there a substantial decrease in family resources allocated to after-school tutoring. If the contributions of both fathers and mothers are more similar and complementary in fostering children’s academic performance compared to other caregivers, such as grandparents, then the absence of both parents can have a more profound effect on a child’s human capital. This scenario warrants greater policy attention than the more commonly considered cases of single-parent absence (5).

Second, there is a significant number of LBC with both parents migrated in rural China. In recent years, the Chinese government has made considerable efforts to enhance public school access for the children of migrant workers [e.g., (10, 11)]. However, national statistics indicate that as of 2018, there were still approximately 7 million LBC in rural China (12) who are cared for by grandparents or no one when both parents are away. Research on their academic performance and overall well-being is essential and warrants further investigation.

The paper proceeds as follows. Section 2 provides the background of LBC and analyzes the effect of both parents migrating on children’s education. Section 3 describes the data. Section 4 introduces our empirical framework and analyzes our empirical results. Section 5 concludes.

## 2 Both parents migrating and its impact on children’s education

In China, the migration of both parents was relatively uncommon before the 1990s. Over the past four decades, China has undergone the most significant rural–urban migration in human history, as noted by Zhao (13). This migration surge can be attributed to the rapid economic growth that commenced in the late 1970s and the substantial disparities between urban and rural areas, which led to hundreds of millions of rural laborers relocating to cities (14). As seen in other developing countries, male individuals dominated rural–urban migration in the 1980s. According to a national community survey-based study documented in Rozelle et al. (15), the rate of off-farm participation for men was eight times higher than that for women during this period. Likewise, Zhang et al. (16) reported that in rural China in 1981, the off-farm participation rate for men (27%) significantly exceeded that for women (4%).

However, the increasing employment opportunities for women have led to both parents migrating becoming a common phenomenon. In the 1990s, the number of female migrants grew at a faster rate than that of male migrants due to the high demand in the labor market and the expansion of female-dominated industries, such as the textile industry, as highlighted by Zhang et al. (16). Consequently, more female laborers migrated to cities. For instance, according to Zhao (13), the percentage of women among rural–urban migrants increased to 28% in Sichuan province by 1995. In the most recent data from the 2013 Investigational and Monitoring Report of Chinese Migrant Workers by the National Bureau of Statistics of China (NSBC), an estimated 161 million rural migrants were employed outside their home areas for periods exceeding six months, with over 130 million being individual migrants who left their rural family members behind (17).

Due to stringent residential policies in destination cities, rural migrants are often compelled to leave their children in their hometowns (18). Unlike many other countries, China enforces rigorous residential policies that make it challenging for rural migrants to establish permanent residency in urban areas (5, 19). Without the requisite local city registration status, these migrants are denied access to public services, particularly in the realm of education. Consequently, the majority of migrant parents are left with no choice but to leave their children in their home townships or villages, resulting in a substantial population of LBC in rural areas (20). According to a report from the All-China Women’s Federation (21), based on data from the 2010 Population Census, there were over 61 million LBC in rural areas, with 47% of them being left by both parents.

With the number of LBC increasing rapidly, the educational performance of LBC has attracted the attention of many scholars. However, the results of their studies are mixed. Some studies suggest that parental migration has a positive effect on children’s educational performance (22–24). Conversely, some other studies argue that parental migration hurts children’s educational performance (5, 6, 25). As a third type standing in opposition to the above findings, some studies have shown that parental migration has no effect on the educational performance of LBC (26, 27).

The inconsistency of findings may be attributed to previous studies’ incomplete estimation of the overall effect of parental migration. Previous studies primarily focus on the direct effect of parental migration, ignoring that there are other significant indirect pathways through which parental migration affects LBC’s educational performance. Without sufficient consideration of these indirect pathways, it is easy to overestimate or underestimate the effect of parental migration.

First, the migration of both parents has negative impacts on the LBC’s academic performance due to a significant reduction in parental involvement in family tutoring (28). Previous studies have indicated that the average time dedicated to family tutoring in cases of both parents migrating is 4.195 min per day, which represents only 29.443% of the time invested by children with at least one parent at home (1). Additionally, LBC tend to receive lower quality of family tutoring compared to non-LBC. Their primary caregivers are more likely to be grandparents, non-parental relatives, or neighbors. Evidence suggests that these caregivers often possess less caregiving-related knowledge, lower levels of formal education and guidance, and spend less time with and provide less supervision to the left-behind child (29, 30).

Second, the migration of both parents can also positively impact children’s academic performance through increased education expenditure (31–33). The migration of both parents significantly increases family income (34, 35), and remittances from their earnings can alleviate household budget constraints, resulting in increased investment in quality education (36–39).

Third, left-behind children also exhibit varying rates of participation in private tutoring and enrollment in boarding schools. Private tutoring serves as a significant avenue for parents wishing to be actively involved in their left-behind children’s education (40). Xu et al. (41) further support this by finding that LBC are more likely to engage in private tutoring. However, the absence of parents from home may have the opposite effect, discouraging them from enrolling their children in private tutoring, as suggested by Zhao and Chen (9). Li and Hu (42) have reported a similar negative impact of parental migration on private tutoring. As such, the nature of the relationship between parental migration and private tutoring participation necessitates further research.

Finally, to the best of our knowledge, there have been no studies that quantitatively estimate the impact of parental migration on the enrollment in boarding schools, which are known to influence children’s academic performance (43, 44). Due to the belief that boarding schools represent an optimal choice for the development of left-behind children, a substantial number of boarding schools have been constructed in rural China (44). However, as far as our knowledge goes, there is a lack of quantitative research analyzing the impact of parental migration on boarding school enrollment.

Therefore, the sign of the overall effect of both parents migrating on the school performance of left-behind children is *a priori* unclear and remains an empirical question. As discussed above, except for the direct negative impact of both parents migrating, the indirect impact through which both parents migrating affects the school performance of left-behind children needs quantitative estimation. If the indirect impact is positive and large enough to offset the negative direct impact, the overall effect of both parents migrating on the education of left-behind children will be positive. If so, the rationality of both parents migrating will be empirically evidenced.

## 3 Data

### 3.1 Data source

Data used in this study are from the China Family Panel Studies (CFPS), which covers 25 provinces/municipalities/autonomous regions (excluding Hong Kong, Macao, Taiwan, Xinjiang, Tibet, Qinghai, Inner Mongolia, Ningxia, and Hainan). The CFPS is a national representative, longitudinal survey of Chinese communities, families, and individuals, launched by the Institute of Social Science Survey (ISSS) of Peking University. The first interview was officially launched in 2010, with follow-up interviews conducted every 2 years thereafter, with cross-wave tracking rates of baseline households are all above 85%.

The CFPS sample is a multi-stage probability sample. The first-stage sample are administrative districts/counties, the second-stage sample are administrative villages/neighborhood committees, and the third-stage sample are households. The sampling in the first two stages is based on official administrative data, employing implicit stratification method to ensure geographical representativeness. In the third stage, an end sampling frame is constructed using the map-address method in the selected sample villages/neighborhood committees, and sample households are drawn using circular isometric sampling with a randomized starting point.

This study focuses on children aged 10–15 living in rural for three reasons. First, these ages are crucial period in the transition from childhood to adulthood, they probably are more vulnerable to parental migration (1). Same age range are used in previous studies, such as Liu et al. (43). Second, according to the Ministry of Civil Affairs, the upper age limit of LBC is 15 years old. Third, the CFPS only collects information on cognitive ability of people aged 10 and above.

The CFPS has two sets of questionnaires, namely Set A and Set B, for assessing children’s cognitive. Set A is used in our study, because Set A are tailored to children’s educational achievements and are more closely aligned with the subject matter under examination in this paper. Set B questions, on the other hand, pertain to children’s potential. Since Set A questions were surveyed in 2010, 2014, and 2018, this study makes use of data spanning these 3 years.

During the survey, sampled children answered both verbal and mathematical problems. Verbal problems consisted 34 Chinese characters, arranged in order of increasing difficulty. The question number corresponding to the most challenging question answered correctly by the respondent is used to determine their verbal score. Likewise, mathematical problems included 24 questions, also organized by difficulty, and the question number of the most challenging question answered correctly by the respondent determines their math score. For additional information about the verbal and mathematical tests, please refer to the official CFPS website.^1^

In addition to raw verbal test scores and raw math test scores, we have created an indicator that reflects the overall cognitive ability. It’s important to note that verbal and math tests are measured on different scales, with the former ranging from 0 to 34 and the latter ranging from 0 to 24. To facilitate comparison and analysis, we standardized the scores using a Z-Score, following the method of previous studies (45). This standardization process ensures that the standardized scores had a mean of 0 and a standard deviation of 1, making them independent of the data’s magnitude and facilitating data comparability (45). However, it’s worth noting that standardization alters the original meaning of the data, allowing for comparisons between data points (46). Consequently, this study also analyzed the raw test scores to maintain the original data’s context.

Table 1 shows the summary statistics for our full sample. The overall mean standardized cognitive ability of the children in our sample is −0.071 (row 1), while the mean of raw cognitive ability is 31.850 (row 2). Among the 34 verbal questions, the average questions corrected answered is 21, with a standardized verbal test score of 0.015 (rows 3 and 4). Less than half of math questions (10.64 vs. 24.00) are corrected answered in our sample and the standardized math test score is 0.055 (rows 5 and 6). It is worth noting that the raw test scores and the standardized scores in Table 1 are consistent with those reported in previous studies [e.g., (43)].

**Table 1:** Descriptive statistics for the full sample.

Variables	Mean	Standard deviation	Min	Max
**Cognitive ability**				
Standardized cognitive ability	−0.071	1.833	−5.153	4.391
Cognitive ability	31.850	10.590	0.000	58.000
Standardized verbal test score	0.015	1.011	−2.858	1.685
Verbal test score	21.290	7.578	0.000	34.000
Standardized math test score	0.055	0.990	−2.513	2.974
Math test score	10.640	4.698	0.000	24.000
**Core independent variables**				
Both parents migrating (yes = 1)	0.252	0.434	0	1
Only paternal migrating (yes = 1)	0.170	0.452	0	1
Only maternal migrating (yes = 1)	0.050	0.501	0	1
**Education**				
Private tutoring dummy (yes = 1)	0.172	0.377	0	1
Private tutoring fee (thousand yuan)	0.166	1.008	0	4.800
Boarding school dummy (yes = 1)	0.359	0.481	0	1
Boarding fee (thousand yuan)	0.069	0.365	0	1.800
Education expenditure (thousand yuan)	1.858	3.051	0	15.000
Key school dummy (yes = 1)	0.144	0.324	0	1
Key class dummy (yes = 1)	0.092	0.270	0	1
**Individual characteristics**				
Family tutoring time (hours/week)	1.904	1.525	0	18.000
Family income (thousand yuan)	43.72	45.25	20	224.000
Verbal fundamentals (excellent/good = 1)	0.401	0.492	0	1
Math fundamentals (excellent/good = 1)	0.508	0.500	0	1
Age (year)	12.60	1.678	10	15.000
Male (yes = 1)	0.522	0.500	0	1
Own educational expectation (college and above = 1)	0.597	0.491	0	1
Parental education (high school and above = 1)	0.112	0.316	0	1
Observations	4,643			

Sources: Authors’ analysis.

LBC constitute 25.2% of our sample, which is larger than those with only one parent migrating (row 7). Of those that experience only one parent migrating, paternal migration is the most common, affecting 17.0% of our sample (row 8). Maternal migration (while the father is at home) is relatively rare, accounting for just 5% of the sample (row 9). The distribution of children with both parents migrating and those with only one parent migrating aligns closely with previous studies, such as Tao and Zhou (5), and Zhang et al. (1).

### 3.2 Difference between LBC and non-LBC

To compare LBC and non-LBC, we categorized the sample into two groups: LBC (with both parents migrating) and non-LBC (at least one parent staying at home). According to Table 2, LBC exhibit statistically significant advantages in terms of cognitive ability compared to non-LBC. The standardized cognitive ability and the raw cognitive ability of LBC are, respectively, 0.231 and 1.021 higher than those of non-LBC (rows 1 and 2). The standardized verbal test score for LBC is 0.129 higher than that of non-left-behind children (row 3), while the raw verbal test score is 0.809 higher (row 4). LBC also demonstrate significantly higher standardized math test scores by 0.101 (row 5), with the math test score being 0.206 higher (row 6).

**Table 2:** Descriptive statistics of sub-sample.

	Non-left-behind Children	Left-behind Children	Difference
	(1)	(2)	(3) = (1)–(2)
**Cognitive ability**			
Standardized cognitive ability	−0.280	−0.050	−0.231^***^
Cognitive ability	30.82	31.84	−1.021^**^
Standardized verbal test score	−0.146	−0.016	−0.129^***^
Verbal test score	20.54	21.35	−0.809^***^
Standardized math test score	−0.135	−0.033	−0.101^***^
Math test score	10.34	10.55	−0.206
**Private tutoring and boarding school**			
Private tutoring dummy (yes = 1)	0.186	0.129	0.056^***^
Boarding school dummy (yes = 1)	0.268	0.647	−0.379^***^
**Individual characteristics**			
Family tutoring time (hours/week)	2.019	0.959	1.061^***^
Family income (thousand yuan)	42.64	51.67	−9.034^***^
Observations	3,473	1,170	

^*^p < 0.1, ^**^p < 0.05, ^***^p < 0.01. Sources: Authors’ analysis.

**Figure 1:**
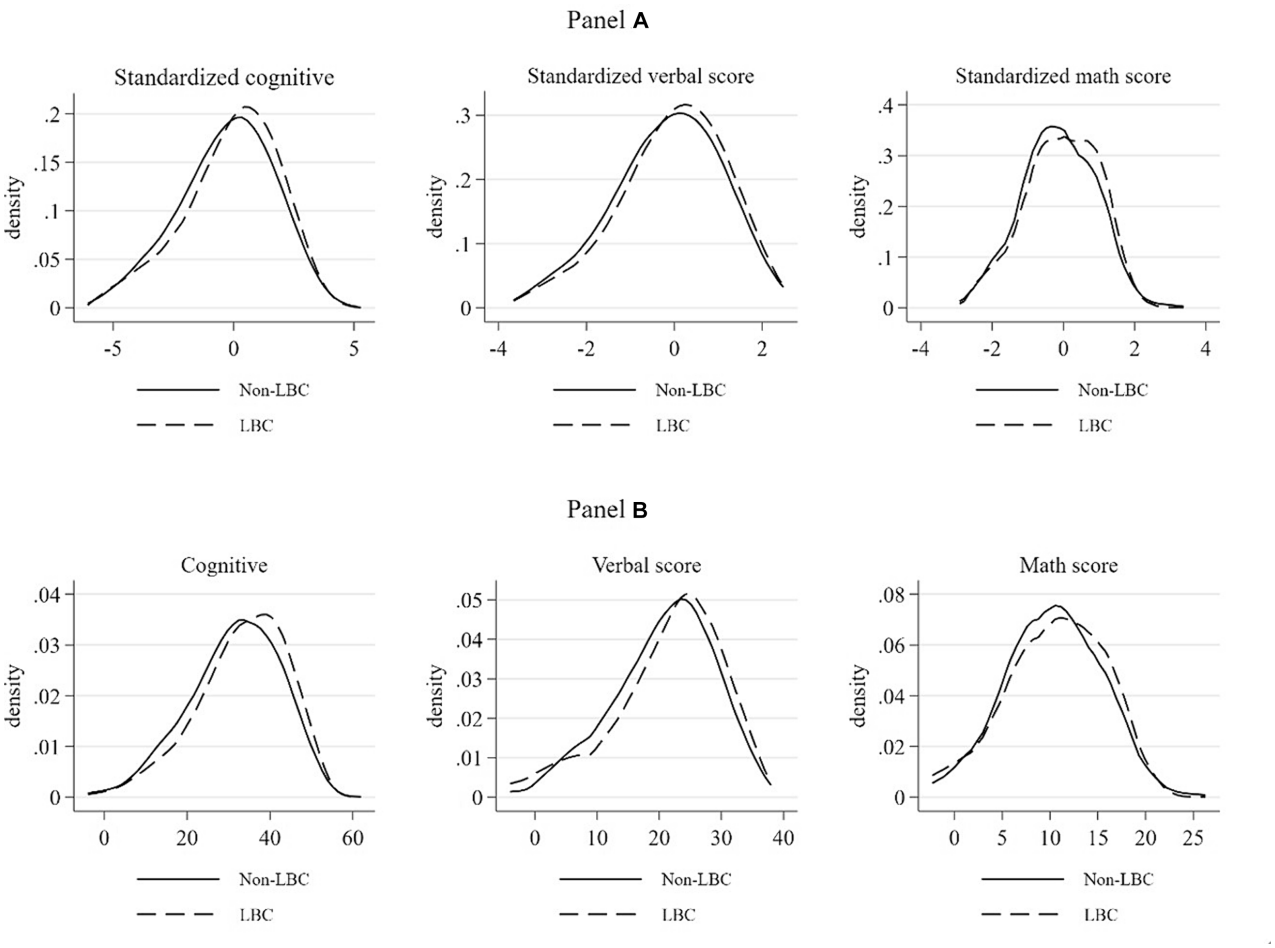
Distribution of various scores of LBC and non-LBC. Sources: Authors’ analysis.

Figure 1 provides a visual representation of the differences between LBC and non-LBC. Panel A illustrates the distribution of standardized data, while Panel B displays the distribution of raw data. In Panel A, it’s evident that the peaks of the LBC distribution are all slightly shifted to the right in comparison to the peaks of the non-LBC distribution. This suggests that, on average, LBC tend to achieve slightly higher scores in cognitive ability, verbal test scores, and math scores compared to non-LBC. Similarly, Panel B reveals a similar pattern. In both the standardized and raw data, it’s clear that the school performance of LBC is relatively better than that of non-LBC.

Upon further examination, it becomes evident that LBC are less inclined to participate in private tutoring and more likely to enroll in boarding schools compared to non-LBC. As indicated in the 7th row, around 18.6% of non-LBC engaged in private tutoring, which is 5% higher than the participation rate among LBC. This finding, though surprising, aligns with previous studies, including Duan et al. (47), which also reported a similar trend. Conversely, the participation rate for boarding schools is more than two times higher for LBC in comparison to non-LBC. This discrepancy is evident in the 8th row, with the participation rate for LBC at 0.647, while for non-LBC it stands at 0.268.

Table 2 provides further insights, revealing that LBC receive less family tutoring than non-LBC, but have higher family income. As expected, when both parents migrate, there is a reduction in the time allocated to tutoring their children, as demonstrated in the 9th row. Specifically, the average family tutoring time for LBC with both parents migrating is 0.959 h per week, whereas non-LBC receive 2.091 h per week, indicating that LBC with both parents migrating receive less tutoring time. Additionally, as shown in the 10th row, the migration of both parents leads to an increase in family income by 9,034 yuan, representing a 21% higher family income compared to non-LBC. These findings shed light on the trade-offs that LBC face in terms of family tutoring and financial resources in the context of parental migration.

## 4 Econometric model and results

### 4.1 Econometric model

One potential issue when estimating the impact of parental migration on children’s cognitive abilities is endogeneity. In this study, endogeneity arises when both parents’ decisions to migrate and their children’s cognitive outcomes are influenced by common factors, such as shared genetics and family culture. For instance, parents with certain aggressive or struggling genetic traits may be more inclined to migrate, and their children may have higher cognitive abilities due to the intergenerational transmission of genes (24). In other words, fathers who are more capable tend to migrate, and their similarly capable children are more likely to remain in school and achieve higher scores (37). This association between both parents migrating and their children’s cognitive abilities can result in a bias that tends to overestimate the impact of parental migration on their children’s cognitive outcomes if the impact is positive.

Furthermore, it is worth noting that families facing poor socio-economic conditions are more likely to migrate if those conditions also have a detrimental impact on their children’s school performance. This tendency can lead to an underestimation of the true impact of parental migration on their children’s cognitive abilities (48). In essence, without a comprehensive understanding of the socio-economic background of the family, it is prone to obtaining biased estimates when examining the effects of parental migration.

To mitigate the potential endogeneity issue to the best extent possible, we employ the Fixed Effects (FE) model, which allows us to control for the endogeneity problem caused by unobserved individual characteristics (48). By incorporating these control measures, we aim to minimize the influence of confounding factors and improve the reliability of our estimates regarding the impact of parental migration on children’s cognitive abilities.

In the FE model, for each child i, we have:

(1) $$Y_{\mathrm{it}}=\beta_{0}+\beta_{1}\mathrm{Migratio}n_{\mathrm{it}}+\beta X_{\mathrm{it}}+u_{i}+Year_{t}+\varepsilon_{\mathrm{it}}$$

Where $Y_{\mathrm{it}}$ denotes children’s cognitive ability. There are six independent variables in our paper: standardized cognitive ability, raw cognitive ability, standardized verbal test score, raw verbal score, standardized math score, and raw math score. $u_{i}$ are the time invariant individual characteristics, *Year_t_* is time dummies, and $\varepsilon_{\mathrm{it}}$ is the error term.

In Equation (1), the variable “*Migration*” is represented as a dummy variable that takes a value of one when both parents of the children migrate. The coefficient associated with this variable, denoted as β, captures the conditional average outcome difference between children classified as LBC (those with both parents migrating) and non-LBC (those without both parents migrating). Therefore, the estimated coefficient β quantifies the direct impact of both parents migrating on children’s school performance. It provides an indication of how children’s educational outcomes are affected when both parents migrate compared to the situation where one or neither parent migrates.

$X_{\mathrm{it}}$ is a matrix of individual, family controls. Individual characteristics include variables such as children’s age and gender, indicator for whether the children wish to obtain university and higher qualifications, indicators for their performance in verbal and math, and indicator for whether the children attend private tutorials. Family characteristics include family tutoring time, family income, family size, and an indicator for whether the highest level of education of the children’s parents is high school or above. In addition, we also control school type and class type, proxies that approximate the quality of public education.

As previously discussed, the impact of both parents migrating extends beyond the direct effect on LBC’s school performance. It can also influence other variables that subsequently affect LBC’s educational outcomes indirectly. These variables include: private tutoring participation, boarding school participation, family tutoring time and family income. By considering these variables, the study aims to examine the indirect pathways through which the migration of both parents can affect LBC’s educational outcomes. Understanding these indirect effects helps provide a more comprehensive understanding of the overall impact of parental migration on LBC’s school performance.

To quantitatively estimate the indirect effects of both parents migrating on children’s school performance, it is necessary to establish a model that captures the impact on the before mentioned indirect channels. Therefore, the following model is set up:

(2) $$Z_{\mathrm{it}}=\gamma_{0}+\gamma_{1}\mathrm{Migratio}n_{\mathrm{it}}+\gamma X_{\mathrm{it}}+u_{i}+Year_{t}+\xi_{\mathrm{it}}$$

In Equation (2), the dependent variable Z comprises four variables: private tutoring, family tutoring, family income, and boarding school participation. Logit models are estimated when private tutoring participation and boarding school participation (both of them are discrete variables) are treated as the dependent variables.

Referring to Lin’s method, the indirect effect of both parents migrating on children’s academic performance can be obtained by multiplying the coefficient β, representing the impact of each indirect pathway on children’s academic performance, by the coefficient $\gamma_{1}$ , representing the impact of both parents migrating on each indirect pathway (49). The sum of direct and indirect effects constitutes the total impact of both parents migrating.

### 4.2 Estimation results

#### 4.2.1 Direct effect of both parents migrating on LBC’s school performance

As mentioned earlier, the coefficient of migration represents the direct effect of both parents migrating on children’s educational outcomes. The estimation results of Equation (1) is shown in Table 3. As shown in the first row of Table 3, the estimated coefficient of both parents migrating is −0.136 in the standardized cognitive equation. This means that the standardized cognitive score of LBC is 0.136 lower than that of non-LBC, indicating a negative direct impact of parental migration on LBC’s cognitive ability. Similar negative estimated coefficients of both parents migrating are found in the raw cognitive ability, standardized/raw verbal scores and standardized/raw math scores, further underscoring the adverse direct impact of parental migration on LBC’s educational outcomes.

**Table 3:** Impact of both parents migration on their children’s school performance.

	Cognitive		Math		Verbal	
	Standardized	Original	Standardized	Original	Standardized	Original
	(1)	(2)	(3)	(4)	(5)	(6)
Both parents migrating (yes = 1)	−0.136^**^	−0.579^*^	−0.109^***^	−0.467^***^	−0.027^*^	−0.112
	(0.06)	(0.33)	(0.03)	(0.13)	(0.02)	(0.14)
Private tutoring dummy (yes = 1)	0.435^***^	2.826^***^	0.205^***^	0.760^***^	0.230^***^	1.804^***^
	(0.06)	(0.43)	(0.04)	(0.15)	(0.04)	(0.31)
Family tutoring time (hours/week)	0.011^*^	0.081^**^	0.005	0.022	0.006	0.061^**^
	(0.01)	(0.04)	(0.00)	(0.02)	(0.00)	(0.03)
Family income (thousand yuan)	0.016^***^	0.080^***^	0.010^***^	0.034^**^	0.007^***^	0.031
	(0.00)	(0.02)	(0.00)	(0.01)	(0.00)	(0.02)
Boarding school dummy (yes = 1)	0.332^***^	2.053^***^	0.175^***^	0.865^***^	0.161^***^	1.230^***^
	(0.06)	(0.38)	(0.03)	(0.13)	(0.03)	(0.27)
Age (year)	0.507^***^	3.017^***^	0.278^***^	1.664^***^	0.231^***^	1.743^***^
	(0.01)	(0.10)	(0.01)	(0.53)	(0.01)	(0.42)
Verbal fundamentals	0.384^***^	2.360^***^	0.137^***^	0.203	0.250^***^	1.781^***^
(excellent/good = 1)	(0.05)	(0.37)	(0.03)	(0.13)	(0.03)	(0.26)
Math fundamentals	0.256^***^	1.485^***^	0.155^***^	0.723^***^	0.109^***^	0.801^***^
(excellent/good = 1)	(0.05)	(0.36)	(0.03)	(0.12)	(0.03)	(0.25)
Own educational expectation	0.297^***^	1.752^***^	0.165^***^	0.440^***^	0.134^***^	0.973^***^
(college and above = 1)	(0.05)	(0.33)	(0.03)	(0.04)	(0.03)	(0.23)
Family size	−0.106^***^	−0.643^***^	−0.035^*^	−0.157^*^	−0.072^***^	−0.498^***^
	(0.03)	(0.22)	(0.02)	(0.09)	(0.02)	(0.16)
Constant	−6.773^***^	−7.293^***^	−3.834^***^	−6.643^***^	−2.969^***^	−0.777
	(0.25)	(1.69)	(0.14)	(0.67)	(0.15)	(1.19)
Individual FE	Yes	Yes	Yes	Yes	Yes	Yes
Year FE	Yes	Yes	Yes	Yes	Yes	Yes
R^2^	0.301	0.257	0.298	0.279	0.217	0.194
Observations	4,643	4,643	4,643	4,643	4,643	4,643

^*^p < 0.1, ^**^p < 0.05, ^***^p < 0.01. Sources: Authors’ analysis.

We believe that there are at least two reasons for the adverse direct impact of parental migration on children’s educational outcomes. First, when both parents migrate, the children left behind tend to receive less parental support and supervision (24). This lack of parental involvement can lead to distraction while studying or provide more opportunities for undesirable behaviors, such as spending extra time on television, computer games or social media, rather than completing homework or focusing on their studies (50, 51). Second, LBC often lack adequate parental care and may be more susceptible to malnutrition, which can impact their energy and ability to concentrate on learning. For example, Mao et al. (25) found parental absence may lead to a decline in children’s overall health and a decrease in their class efforts, further affecting their educational outcomes.

It is important to emphasize that the first row of Table 3 reflects the direct impact of both parents migrating on children’s school performance. However, there are other significant indirect pathways through which parental migration affects left-behind children, including private tutoring, family tutoring, family income, and boarding schools, as illustrated in Table 3. Table 3 reveals that all the indirect pathways positively impact children’s cognitive abilities (rows 2 to 5). Therefore, to assess the overall impact of both parents migrating on LBC’s school performance, it is essential to consider the indirect effects as well.

#### 4.2.2 Indirect effect of both parents migrating on LBC’s school performance

The estimation results of Equation (2) are shown in Table 4. The first noteworthy finding in Table 4 is that the LBC are less likely to attend private tutoring classes (column 1). There are several potential reasons for the lower participation rate of private tutoring among LBC. First, LBC often need to allocate more time to farm work (28, 37, 52) or household chores due to the lack of available labor at home. Second, LBC are primarily under the care of their grandparents, who may not prioritize their education as much as their parents would and not actively encourage the children to participate in private tutoring classes (47). Third, rural areas often lack well-developed public transportation (53). When both parents are working outside the home, there may be no one available to facilitate their transportation to these classes.

**Table 4:** Impact of both parents migration on private tutoring, family tutoring, family income, and boarding school participation.

	Private tutoring dummy	Family tutoring time	Family income	Boarding school dummy
	(1)	(2)	(3)	(4)
Both parents migrating (yes = 1)	−0.044^***^ (0.01)	−0.996^***^ (0.14)	13.246^***^ (2.19)	0.280^***^ (0.01)
Private tutoring dummy (yes = 1)		0.710^***^ (0.16)	5.517^*^ (2.89)	−0.050^***^ (0.02)
Family tutoring time (hours/week)	0.043^***^ (0.01)		−1.352^***^ (0.45)	−0.014^***^ (0.00)
Family income (thousand yuan)	0.009^***^ (0.00)	0.015^***^ (0.01)		0.000 (0.00)
Boarding school dummy (yes = 1)	−0.271^***^ (0.10)	−0.871^***^ (0.14)	2.820^*^ (1.50)	
Age (years)	0.051^*^ (0.03)	−0.447^***^ (0.03)	3.953^***^ (0.30)	0.073^***^ (0.00)
Verbal fundamentals (excellent/good = 1)	0.256^***^ (0.10)	−0.354^***^ (0.13)	−8.796^***^ (3.29)	0.001 (0.02)
Math fundamentals (excellent/good = 1)	0.131 (0.10)	0.305^**^ (0.13)	3.230 (2.92)	0.005 (0.01)
Own educational expectation (college and above = 1)	0.290^***^ (0.09)	0.112 (0.12)	2.273 (2.89)	0.011 (0.01)
Family size	−0.133^***^ (0.03)	0.103 (0.08)	3.388 (3.36)	−0.015^***^ (0.00)
Constant		6.518^***^ (0.61)	28.665^*^ (15.17)	
Individual FE	Yes	Yes	Yes	Yes
Year FE	Yes	Yes	Yes	Yes
R^2^		0.1686	0.0987	
Observations	4,643	4,643	4,643	4,643

^*^p < 0.1, ^**^p < 0.05, ^***^p < 0.01. Sources: Authors’ analysis.

The second notable finding in Table 4 is that LBC receive significantly less family tutoring (column 2). The estimation results indicate that when both parents migrate, it results in a reduction of approximately 50% in family tutoring time. Parents typically play a crucial role as the primary tutors for their children within the family. Therefore, when both parents migrate, LBC are primarily cared for by their grandparents, who may have limited knowledge (54) and cannot provide the same level of supervision and tutoring as the children’s parents.

The third significant finding in Table 4 reveals that both parents’ migration results in increased family income. This aligns with the theory proposed by Stark and Bloom (55) in “The New Economics of Labor Migration,” which suggests that migration decisions are often made collectively to diversify risks and maximize household economic welfare. Similarly, in rural China, a significant portion of migrants’ incomes is dedicated to remittances (48). In line with this, the study shows that both parents migrating increases family income by 13,246 yuan, representing 31% of the family’s income.

Lastly, Table 4 also indicates that LBC are more likely to enroll in boarding schools (column 4), which is consistent with previous research (43). Boarding schools offer a stable living environment with dedicated staff to care for and supervise students, providing an improved learning environment, especially for LBC (56, 57).

#### 4.2.3 Total effect of both parents migrating on LBC’s school performance

In this section, we will quantitatively assess the overall impact of both parents migrating on LBC’s school performance. As discussed earlier, while the direct impact is negative (as seen in row 1 of Table 3), the overall impact remains unclear. Table 4 reveals that both parents migrating have a significant impact on private tutoring, family tutoring, family income, and boarding school participation. On the other hand, private tutoring, family tutoring, family income, and boarding school participation significantly affect LBC’s school performance, as demonstrated in rows 2–5 of Table 3. This section aims to evaluate the combined contributions of the direct and indirect impacts of both parents migrating on children’s cognitive ability.

Table 5 provides an overview of the total effects of both parents’ migration on children’s school performance, drawing from estimates in Tables 3, 4. The first column in Table 5 showcases the estimated coefficients from Table 3, representing the influence of each factor on the child’s school performance. The second column of Table 5 contains the estimated coefficients from the first row of Table 4, indicating the impact of both parents migrating on each factor. By multiplying the values in the first and second columns, we can determine the effect of both parents’ migration on children’s school performance through various factors, as presented in column 3 of Table 5. The fourth column illustrates the absolute value of the contribution of the impact of both parents migrating to changes in the child’s cognitive ability through various factors. Finally, the last column demonstrates these contributions as percentages.

**Table 5:** Summary of marginal effects.

	Estimated coefficient (from Table 3)	Estimated coefficient (from Table 4)	Impact (3) = (1) × (2)	Contribution (4) = (3)/Total change*100%
	(1)	(2)	(3)	(4)
**Panel A: Standardized cognitive ability (0.231)**				
Both parents migrating	−0.136	1.00	−0.136	−58.874
Private tutoring dummy	0.435	−0.044	−0.019	−8.225
Family tutoring time	0.011	−0.996	−0.011	−4.762
Family income	0.016	13.246	0.212	91.775
Boarding school dummy	0.332	0.280	0.093	40.260
Total effects			0.139	60.173
**Panel B: Non-standardized cognitive abilities (1.021)**				
Both parents migration 1	−0.579	1.00	−0.579	−56.709
Private tutoring dummy	2.826	−0.044	−0.124	−12.145
Family tutoring time	0.081	−0.996	−0.081	−7.933
Family income	0.080	13.246	1.060	103.820
Boarding school dummy	2.053	0.280	0.575	56.317
Total effects			0.851	83.350
**Panel C: Standardized math scores (0.101)**				
Both parents migration 1	−0.109	1.00	−0.109	−107.921
Private tutoring dummy	0.205	−0.044	−0.009	−8.911
Family tutoring time	0.005	−0.996	−0.005	−4.950
Family income	0.010	13.246	0.132	130.693
Boarding school dummy	0.175	0.280	0.049	48.515
Total effects			0.058	57.426
**Panel D: Non-standardized math scores (0.206)**				
Both parents migration 1	−0.467	1.00	−0.467	−226.699
Private tutoring dummy	0.760	−0.044	−0.033	−16.019
Family tutoring time	0.022	−0.996	−0.022	−10.680
Family income	0.034	13.246	0.450	218.447
Boarding school dummy	0.865	0.280	0.242	117.476
Total effects			0.17	82.524
**Panel E: Standardized Chinese scores (0.129)**				
Both parents migration 1	−0.027	1.00	−0.027	−20.930
Private tutoring dummy	0.230	−0.044	−0.010	−7.752
Family tutoring time	0.006	−0.996	−0.006	−4.651
Family income	0.007	13.246	0.093	72.093
Boarding school dummy	0.161	0.280	0.045	34.884
Total effects			0.095	73.643
**Panel F: Non-standardized Chinese scores (0.809)**				
Both parents migration 1	−0.112	1.00	−0.112	−13.844
Private tutoring dummy	1.804	−0.044	−0.079	−9.765
Family tutoring time	0.061	−0.996	−0.061	−7.540
Family income	0.031	13.246	0.411	50.803
Boarding school dummy	1.230	0.280	0.344	42.522
Total effects			0.503	62.176

Sources: Authors’ analysis.

Panel A of Table 5 reveals that the overall impact of both parents’ migration has a positive effect on LBC’s standardized cognitive ability, despite the direct impact being negative. As indicated in the first row, the direct influence of both parents’ migration accounts for approximately −59% of the variation (row 1). Conversely, the indirect influence of both parents’ migration contributes approximately −8% through the private tutoring channel, −5% through the family tutoring channel, 92% through the family income channel, and 40% through the boarding school channel (rows 2–5). This means that the negative direct impact of both parents’ migration can be completely offset by the positive indirect impact through the increase in family income. When considering both the direct and indirect effects, both parents’ migration leads to a substantial 60% increase in standardized cognitive abilities for LBC.

The analysis of children’s raw cognitive abilities yields consistent findings, as demonstrated in Panel B. The negative direct impact of both parents migrating (−57%) is outweighed by the positive indirect influence through enhanced family income (104%). Furthermore, when considering the cumulative effect of the other indirect factors (i.e., private tutoring, family tutoring, and boarding school participation), the total impact is also positive (−12–8% + 56% = 36%). Consequently, the raw cognitive ability of LBC surpasses that of non-LBC by 83%.

Applying the same methodology, we proceed to assess the direct and indirect effects of both parents migrating on math and verbal test scores. Panels C-F display the consistent results, indicating that both parents migrating has a positive and significant total impact on both math and verbal test scores.

It’s essential to highlight that while the negative direct impact of both parents migrating on cognitive abilities aligns with previous research, the overall positive total impact sets this study apart. As demonstrated in Panel A of Table 6, earlier studies consistently found negative direct effects of both parents migrating on children’s math and verbal test scores, which aligns with the findings of this study. However, the contrast emerges when considering the total impact, which encompasses both the direct and indirect effects of both parents migrating. Previous studies reported a negative total impact on cognitive abilities, encompassing verbal and math test scores (Panel B, Table 6). In contrast, this study reveals a significant and positive total impact on cognitive abilities, verbal test scores, and math test scores for LBC.

**Table 6:** Comparison of results from different literature.

	Literature	Dependent variable	Impact of both parents migration
Panel A: Direct impact	This article	Standardized math test score	−0.098
	Zhang et al. (1)		−0.088
	Wu et al. (58)		−0.059
	Chang et al. (26)		Insignificant
	Tao and Zhou (5)		−0.200
	Li et al. (42)		−0.16
	Liu et al. (43)		Insignificant
	Chen et al. (6)		Insignificant
	Zhao et al. (3)		−0.0155
	This article	Standardized verbal test score	−0.060
	Chen et al. (6)		Insignificant
	Zhang et al. (1)		−0.079
	Wu et al. (58)		−0.078
	Tao and Zhou (5)		−0.220
	Liu et al. (43)		Insignificant
	This article	Comprehensive cognitive ability	0.100
Panel B: Total impact	Chen et al. (6)		Negative
	This article	Standardized verbal test score	0.045
	Tao and Zhou (5)		Negative
	This article	Standardized math test score	0.042
	Tao and Zhou (5)		Negative

Sources: Authors’ analysis.

The variance in findings between this study and the existing body of research can be attributed to the inclusion of additional channels through which both parents migrating affects LBC’s school performance. Prior studies, such as Tao and Zhou (5), solely concentrated on the indirect impact through family income. Likewise, Chen et al. (6) solely considered the indirect effects of family income and family tutoring. In contrast, this paper explores multiple indirect impact pathways, encompassing private and family tutoring, family income, and boarding school participation. By considering these additional factors, a more comprehensive understanding of the influences on LBC’s school performance is achieved, which results in differing findings compared to previous studies.

#### 4.2.4 Robustness check

As discussed earlier, the study initially categorized children into two groups: children with both parents migrating (i.e., LBC) and children without both parents migrating (i.e., non-LBC). To enhance the robustness of the results and provide a more comprehensive analysis, we have reclassified all the children into three distinct groups: children with both parents migrating, children with only one parent migrating, and children with both parents staying at home. This reclassification enables a more nuanced examination of the impact. We subsequently re-estimated Equations (1, 2) accordingly, and the detailed estimation results are available upon request. Following the re-estimations, we recalculated both the direct and indirect impacts, and for the sake of simplicity, we will provide a summary of these impacts.

The estimation results validate the robustness of the findings and their insensitivity to the definition of LBC. Panels A and B of Table 7 reveal that the cognitive abilities of left-behind children, whether with both parents migrating or only one parent migrating, are higher than those of children with both parents at home. Furthermore, the difference in cognitive abilities between LBC and children with both parents at home (0.312) is larger than the difference in cognitive abilities between children with only one parent migrating and children with both parents at home (0.113). Similar patterns are observed for verbal and math test scores, as demonstrated in Panels C-F.

**Table 7:** Summary of marginal effects (both parents migrating vs. one parent migrating).

	Estimated coefficient	Estimated coefficient	Impact (3) = (1) × (2)	Contribution (4) = (3)/Total change *100%
	(1)	(2)	(3)	(4)
**Panel A: Standardized cognitive ability (both parents migration vs. both parents at home, 0.312)**				
Both parents migrating	−0.140	1.000	−0.140	−44.87
Private tutoring dummy	0.428	−0.039	−0.017	−5.45
Family tutoring time	0.012	−0.696	−0.008	−2.56
Family income	0.020	15.302	0.306	98.08
Boarding school dummy	0.409	0.292	0.119	38.14
Total effects			0.260	83.33
**Panel B: Standardized cognitive ability (only one parent migration vs. both parents at home, 0.066)**				
Both parents migrating	−0.050	1.000	−0.050	−75.76
Private tutoring dummy	0.428	0.004(insignificant)	0	0.00
Family tutoring time	0.012	0.057(insignificant)	0	0.00
Family income	0.020	8.659	0.173	262.12
Boarding school dummy	0.409	0.034	0.014	21.21
Total effects			0.137	207.58
**Panel C: Standardized math scores (both parents migration vs. both parents at home, 0.142)**				
Both parents migrating	−0.120	1.000	−0.120	−84.51
Private tutoring dummy	0.207	−0.039	−0.008	−5.63
Family tutoring time	0.007	−0.696	−0.005	−3.52
Family income	0.015	15.302	0.230	161.97
Boarding school dummy	0.222	0.292	0.065	45.77
Total effects			0.162	114.08
**Panel D: Standardized math scores (only one parent migration vs. both parents at home, 0.041)**				
Both parents migrating	−0.061(insignificant)	1.000	0	0.00
Private tutoring dummy	0.207	0.004(insignificant)	0	0.00
Family tutoring time	0.007	0.057(insignificant)	0	0.00
Family income	0.015	8.659	0.130	317.07
Boarding school dummy	0.222	0.034	0.008	19.51
Total effects			0.138	336.59
**Panel E: Standardized verbal scores (both parents migration vs. both parents at home,0.169)**				
Both parents migrating	−0.030	1.000	−0.030	−17.75
Private tutoring dummy	0.226	−0.039	−0.009	−5.33
Family tutoring time	0.005	−0.696	−0.003	−1.78
Family income	0.006	15.302	0.092	54.44
Boarding school dummy	0.189	0.292	0.055	32.54
Total effects			0.105	62.13
**Panel F: Standardized Verbal scores (only one parent migration vs. both parents at home,0.025)**				
Both parents migrating	−0.014(insignificant)	1.000	0	0.00
Private tutoring dummy	0.226	0.004(insignificant)	0	0.00
Family tutoring time	0.005	0.057(insignificant)	0	0.00
Family income	0.006	8.659	0.052	208.00
Boarding school dummy	0.189	0.034	0.006	24.00
Total effects			0.058	232.00

Sources: Authors’ analysis.

Additionally, to enhance the robustness of the results and consider the quality of private tutoring and boarding schools, we re-estimated Equations (1, 2) using tutoring fees and boarding fees instead of private tutoring and boarding school dummies. For simplicity, we will provide a summary of the direct and indirect impacts. Similar to the results in Table 5, Panels A and B of Table 8 confirm that LBC have higher cognitive abilities than Non-LBC. These patterns are consistently observed for verbal and math test scores in Panels C–F. In Panels C–F, it is evident that both parents migrating has a positive and significant total impact on both math and verbal test scores.

**Table 8:** Summary of marginal effects (tutoring fees, boarding fees).

	Estimated coefficient	Estimated coefficient	Impact (3) = (1) × (2)	Contribution (4) = (3)/Total change*100%
	(1)	(2)	(3)	(4)
**Panel A: Standardized cognitive ability (0.231)**				
Both parents migrating	−0.130	1.00	−0.130	−56.277
Tutoring fees	0.154	−0.179	−0.028	−12.121
Family tutoring time	0.041	−0.734	−0.030	−12.987
Family income	0.020	11.764	0.235	101.732
Boarding fees	0.393	0.046	0.018	7.792
Total effects			0.065	28.139
**Panel B: Non-standardized cognitive abilities (1.021)**				
Both parents migrating	−0.684	1.00	−0.684	−66.993
Tutoring fees	1.004	−0.179	−0.180	−17.630
Family tutoring time	0.221	−0.734	−0.162	−15.867
Family income	0.196	11.764	2.306	225.857
Boarding fees	2.154	0.046	0.099	9.696
Total effects			1.379	135.064
**Panel C: Standardized math scores (0.101)**				
Both parents migrating	−0.086	1.00	−0.086	−85.149
Tutoring fees	0.091	−0.179	−0.016	−15.842
Family tutoring time	0.024	−0.734	−0.018	−17.822
Family income	0.011	11.764	0.129	127.723
Boarding fees	0.193	0.046	0.009	8.911
Total effects			0.018	17.822
**Panel D: Non-standardized math scores (0.206)**				
Both parents migrating	−0.445	1	−0.445	−216.019
Tutoring fees	0.263	−0.179	−0.047	−22.816
Family tutoring time	0.119	−0.734	−0.087	−42.233
Family income	0.034	11.764	0.623	302.427
Boarding fees	0.804	0.046	0.037	17.961
Total effects			0.081	39.320
**Panel E: Standardized Chinese scores (0.129)**				
Both parents migration	−0.028(insignificant)	1.00	0.000	0.000
Tutoring fees	0.063	−0.179	−0.011	−5.340
Family tutoring time	0.017	−0.734	−0.012	−5.825
Family income	0.008	11.764	0.094	45.631
Boarding fees	0.201	0.046	0.009	4.369
Total effects			0.08	38.835
**Panel F: Non-standardized Chinese scores (0.809)**				
Both parents migrating	−0.388(insignificant)	1.00	0.000	0.000
Tutoring fees	0.467	−0.179	−0.084	−10.383
Family tutoring time	0.133	−0.734	−0.098	−12.114
Family income	0.046	11.764	0.541	66.873
Boarding fees	1.282	0.046	0.059	7.293
Total effects			0.418	51.669

Sources: Authors’ analysis.

## 5 Conclusion

Using data from the CFPS, this study employs a fixed-effects model to address potential estimation biases arising from the endogeneity of children’s left-behind status. It aims to analyze both the direct and indirect impacts of both parents’ absence on the educational performance of LBC. The study confirms that both parents’ migration has a negative direct impact on children’s academic performance, which aligns with prior research findings (1, 5, 58). However, the negative direct impact can be fully offset by the indirect impact channels, such as private tutoring, family tutoring, family income, and boarding school participation. In contrast to previous studies [e.g., (5, 6)], this research reveals a positive overall impact of both parents’ migration on LBC’s school performance.

The findings of this study have importantly policy implications. First, it underscores the need for governments to address the negative consequences of both parents’ migration. As demonstrated in this research, both parents’ migration not only leads to direct negative impacts but also results in negative consequences through reduced family tutoring time and private tutoring participation. Moreover, it should be acknowledged that LBC endure other adverse effects of parental migration, including psychological and mental impacts (59). Based on the study’s findings, reforming the household registration system, especially in cities with a high number of rural–urban migrants, could facilitate these children’s access to public education in destination cities.

Second, policies should be designed to amplify positive effects and mitigate negative consequences arising from both parents’ migration. For instance, as shown in this study, boarding school participation can effectively enhance children’s school performance. Therefore, government efforts should focus on increasing the number and enhancing the quality of boarding schools to counteract the negative impact resulting from the lack of parental care and supervision. Similarly, enhancing the quality of after-school services can assist LBC facing learning difficulties, thus alleviating the disadvantages associated with lower private tutoring participation and reduced family tutoring time.

This study still has some limitations. Firstly, CFPS only conducts cognitive ability tests on samples aged 10 and above, so the age range of LBC studied in this paper is 10–15 years old. We cannot investigate LBC who are younger, although the impact of both parents migrating may be greater on them. Secondly, this paper only analyzes the impact of both parents migrating on the cognitive abilities of LBC. Further research is needed to determine whether considering the impact on the non-cognitive abilities of left-behind children simultaneously would affect the conclusions of this paper.

## Data availability statement

Publicly available datasets were analyzed in this study. This data can be found at: http://www.isss.pku.edu.cn/cfps/download.

## Ethics statement

Ethical review and approval was not required for the study on human participants in accordance with the local legislation and institutional requirements. Written informed consent from the patients/participants or patients/participants legal guardian/next of kin was not required to participate in this study in accordance with the national legislation and the institutional requirements.

## Author contributions

MY: Conceptualization, Data curation, Formal analysis, Investigation, Methodology, Project administration, Resources, Software, Supervision, Validation, Visualization, Writing – original draft, Writing – review & editing.

## Footnotes

^1^See the official CFPS website for details: http://www.isss.pku.edu.cn/cfps/cjwt/wjsjjxxcj/1356846.htm.
